# Endopeptidase-Mediated Beta Lactam Tolerance

**DOI:** 10.1371/journal.ppat.1004850

**Published:** 2015-04-17

**Authors:** Tobias Dörr, Brigid M. Davis, Matthew K. Waldor

**Affiliations:** 1 Division of Infectious Diseases, Brigham and Women’s Hospital and Howard Hughes Medical Institute, Boston, Massachusetts, United States of America; 2 Department of Microbiology and Immunobiology, Harvard Medical School, Boston, Massachusetts, United States of America; University of Washington, UNITED STATES

## Abstract

In many bacteria, inhibition of cell wall synthesis leads to cell death and lysis. The pathways and enzymes that mediate cell lysis after exposure to cell wall-acting antibiotics (e.g. beta lactams) are incompletely understood, but the activities of enzymes that degrade the cell wall (‘autolysins’) are thought to be critical. Here, we report that *Vibrio cholerae*, the cholera pathogen, is tolerant to antibiotics targeting cell wall synthesis. In response to a wide variety of cell wall- acting antibiotics, this pathogen loses its rod shape, indicative of cell wall degradation, and becomes spherical. Genetic analyses revealed that paradoxically, *V*. *cholerae* survival via sphere formation required the activity of D,D endopeptidases, enzymes that cleave the cell wall. Other autolysins proved dispensable for this process. Our findings suggest the enzymes that mediate cell wall degradation are critical for determining bacterial cell fate - sphere formation vs. lysis – after treatment with antibiotics that target cell wall synthesis.

## Author Summary

Inhibition of bacterial cell wall synthesis by antibiotics such as penicillin can lead to unbalanced activity of a poorly defined set of lytic enzymes, termed ‘autolysins,’ which degrade the cell wall and typically cause cell lysis. Here, we report that in *Vibrio cholerae* (the cause of cholera), inhibition of cell wall synthesis results in the formation of viable spheres rather than cell lysis. Paradoxically, sphere formation requires the activity of cell wall degradative enzymes. Inhibition of cell wall synthesis in additional pathogens also leads to sphere formation. These findings expand our understanding of the cellular responses to cell wall acting antibiotics, demonstrating that cell wall degradative enzymes not only function as autolysins, but can also mediate cell survival in the face of cell wall insufficiency.

## Introduction

Nearly all bacteria are surrounded by a rigid cell wall, a structure that maintains cell shape and ensures cellular integrity in the face of potentially extreme osmotic stresses in the environment. The principal component of the cell wall is peptidoglycan (PG), a complex polymer that consists of a polysaccharide web with cross linked peptide sidechains found outside of the cytoplasmic membrane. PG biosynthesis is a multi-step process that begins in the cell cytoplasm, where precursor molecules are built [1]. Once precursors are exported outside the cell membrane, they are assembled into PG by Penicillin Binding Proteins (PBPs), enzymes that catalyze the polymerization of polysaccharide chains and crosslinking of peptide sidechains. Beta lactam antibiotics (penicillins, cephalosporins and carbapenems), which are among the most important antibiotics in current use, covalently bind to and inactivate PBPs [2]. PG’s importance for bacterial survival becomes evident when its synthesis is inhibited by beta lactams or antibiotics that block earlier steps in cell wall synthesis—cells routinely lyse.

It was initially hypothesized that beta lactam-induced lysis was caused by the mechanical force generated by increased turgor pressure that arose upon cessation of PG expansion while the cell maintained other cell growth programs. However, studies in both Gram- positive and Gram-negative organisms indicate that lysis is mediated by enzymatic activity [3,4]. PG cleavage mediated by cell wall hydrolases, also known as autolysins, is presumed to be excessive and/or dysregulated in the absence of ongoing PG synthesis, and the resulting breaches in the cell wall are thought to lead to lysis. Most bacteria contain multiple copies of at least 3 classes of potential autolysins—amidases, lytic transglycosylases and endopeptidases—and all 3 ordinarily play important roles in PG homeostasis [5–8]. An accumulation of degradation products from these enzymes were detected in *Escherichia coli* cells treated with beta lactam antibiotics [9], consistent with the possibility that lysis after inhibition of cell wall synthesis may be associated with the activity of multiple autolysins. However, multiple autolysins are not always important for beta lactam-induced lysis; e.g., in *Streptococcus pneumoniae*, deletion of a single amidase (Atl) renders this gram-positive pathogen completely tolerant to beta lactam-induced lysis [3].

In *E*. *coli*, beta lactam-induced lysis usually starts from the cell septum [10,11], suggesting that amidases, which are recruited to and activated at the site of cell division, might initiate PG cleavage associated with lysis. Supporting this idea, deletion of multiple amidases leads to a lower rate of lysis after exposure to beta-lactam antibiotics [10,12]. In contrast, there is contradictory evidence regarding the role of lytic transglycosylases in the lysis process. Mutants lacking multiple lytic transglycosylases are typically more susceptible to beta lactam antibiotics [13,14], suggesting that these enzymes promote, rather than impair, survival after inhibition of cell wall synthesis. However, overexpression of bifunctional PBPs containing an inactive transpeptidase active site, which mimics exposure to beta lactam antibiotics, results in *E*. *coli* lysis via a process that is largely dependent on LTGs [15]. None of the other predicted cell wall lytic enzymes in *E*. *coli* have been definitively linked to beta lactam-induced lysis. Efforts to define the full set of gene products that mediate bacterial lysis after inhibition of cell wall synthesis or the relative importance of their activities have been thwarted by the fact that the observed phenotype (lysis) is typically rapid, potentially masking differences between mutants, and that most lytic enzymes are highly redundant.

Likely because of the prevalence of cell-wall acting antibiotics in their natural habitats [16], bacteria employ multiple strategies to cope with the dangers associated with inhibition of cell wall synthesis. The most well-studied of these strategies is resistance e.g. by beta lactamases, which inactivate beta lactams. A more passive strategy is dormancy (e.g., formation of persister cells), which allows cells to survive exposure to any normally lethal antibiotic. Persistence is mediated by activation of multiple toxin-antitoxin modules [17,18], which stop growth of a small fraction of bacterial populations and thus confer tolerance to antibiotics that are only active on growing cells [19]. Bacteria that are not replicating due to reaching high cell densities also tend to be tolerant to cell wall-acting antibiotics [20] as do bacteria exposed to factors thought to stabilize the outer membrane [11]. It is unclear what other strategies might exist to survive exposure to cell wall synthesis inhibitors.

Here, we report that *Vibrio cholerae*, the causative agent of the diarrheal disease cholera, routinely tolerates antibiotic-induced inhibition of cell wall synthesis. Similar to most bacteria, *V*. *cholerae* loses the structural integrity of its cell wall following exposure to a wide variety of cell wall synthesis inhibitors. However, in contrast to many other bacteria, this treatment results in formation of viable (though non-dividing) spherical cells, rather than cell lysis. Surprisingly, genetic analyses revealed that *V*. *cholerae* sphere formation depends on the activity of M23 family endopeptidases that are required for cell elongation under conditions of normal growth; in contrast, its amidase and lytic transglycosylases are not required for formation of viable spheres. Furthermore, we found that other important pathogens, including *Pseudomonas aeruginosa* and *Acinetobacter baumannii*, also fail to respond to beta lactam exposure with lysis under certain growth conditions, suggesting that intrinsic, population-wide beta lactam tolerance may be more widespread than currently appreciated.

## Results and Discussion

### *Vibrio cholerae* is highly tolerant to cell-wall acting antibiotics

**Fig 1:**
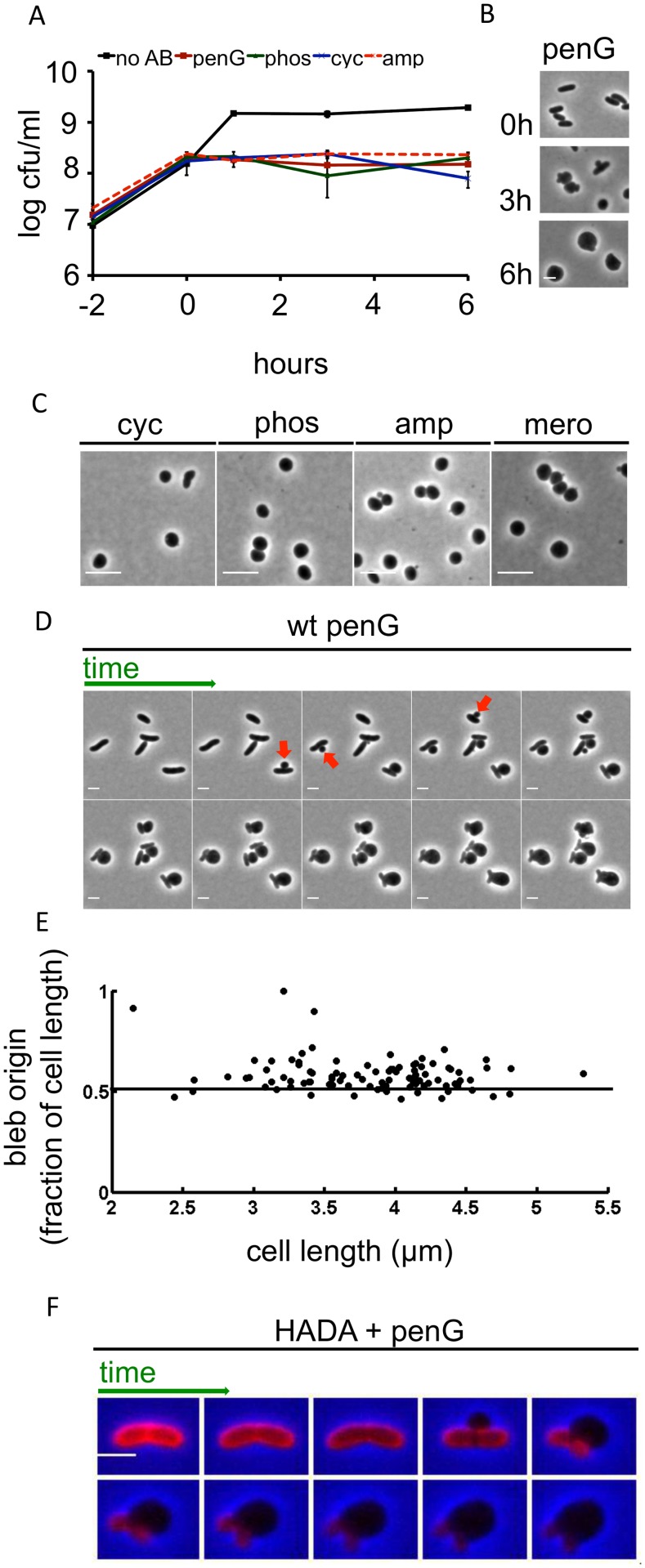
Inhibition of cell-wall synthesis in *V*. *cholerae* leads to sphere formation. (A) Kinetics of viable *V*. *cholerae* cell counts after cells were exposed to various antibiotics that inhibit cell wall synthesis. Cells were grown to ~ 2–3 x 10^8^ cfu/ml and then exposed to 100 μg/ml of penicillin G (penG), phosphomycin (phos), D-cycloserine (cyc), ampicillin (amp) or no antibiotic (no AB) (T0). Data shown are averages of two independent experiments; error bars represent standard deviation. (B) Images of *V*. *cholerae* cells at different time points after exposure to penicillin G from the experiment shown in (A). (C) Morphology of wt exponential phase cells exposed to 100 μg/ml cyc, phos, amp or 1 μg/ml meropenem (mero) for 3 h. (D) Time lapse images of wt cells plated on an agarose pad containing 100 μg/ml pen G. Frames are 5 min apart, scale bar = 2 μm. Arrowheads point to selected blebs. (E) Localization of blebs after exposure to pen G. Three different time lapse series with ~ 40 cells each were obtained as described in (D) and bleb location as the ratio of distance from an arbitrarily chosen pole divided by cell length was measured using ImageJ software. (F) Time lapse images of wt cells stained with the fluorescent D amino acid analogue HADA (50 μM) for 30 min and washed 2 x to remove excess dye prior to imaging on an agarose pad containing pen G. Images were brightness/contrast-adjusted to compensate for photobleaching. Frames are 5 min apart, scale bar = 2 μm. HADA stain is false-colored in red.

We observed that mid- to late exponential phase cultures of *V*. *cholerae* treated with high doses of penicillin G or ampicillin (100 μg/ml, 20 x MIC) failed to divide, but did not show a decline in viable cells (i.e., cfu) (Fig 1A, S1A Fig). Similarly, inhibition of early steps in PG synthesis by D-cycloserine, an inhibitor of D-Ala-D-Ala ligase (100 μg/ml, 2 x MIC), or phosphomycin, an inhibitor of MurA (100 μg/ml, 2x MIC), did not appreciably affect the survival of *V*. *cholerae*. Thus, although antibiotics targeting cell wall synthesis are effective in preventing *V*. *cholerae* proliferation, they do not induce the cell death typically observed in dividing cells of other species. Due to *V*. *cholerae’s* “tolerance” of these chemotherapeutic agents, their effects are not irreversible.

In liquid medium, *V*. *cholerae* lost its rod-shape and eventually assumed a spherical morphology after exposure to the previously mentioned antibiotics or to meropenem (10 μg/ml, 100x MIC) (Fig 1B and Fig 1C). Thus, inhibition of *V*. *cholerae* cell wall synthesis results in the loss of PG’s ‘exoskeletal’ function to maintain cell shape. This is reminiscent of so-called L-forms (artificially-induced cell wall deficient bacteria, [21,22]); however, while L-forms proliferate in the absence of a functional cell wall, *V*. *cholerae* spheres did not divide in the presence of antibiotics (Fig 1A). Moreover, *E*. *coli* L-form generation requires the use of osmotically stabilizing media (e.g., containing high concentrations of sucrose) while *V*. *cholerae* survived exposure to cell wall acting antibiotics in diverse media lacking stabilizing agents, such as LB broth and rabbit cecal fluid (see below).

*V*. *cholerae* sphere formation appears to be independent of which step in cell wall synthesis is inhibited, since a variety of cell wall synthesis inhibitors yielded spheres. Importantly, penicillin also induced formation of viable, spherical *V*. *cholerae* in cecal fluid that was collected from infant rabbits with cholera-like diarrhea (S1B Fig), demonstrating that *V*. *cholerae’s* absence of lysis in response to inhibitors of cell wall synthesis is not due to stabilizing agents present in artificial growth medium, but instead has *in vivo* relevance. Sphere formation typically initiated with blebbing from the midcell (Fig 1D), although we did observe rare instances (~ 3% of cells) where blebbing started closer to the cell poles (Fig 1D and 1E). As blebs became large, the remainder of the cell became smaller, until only the poles of the original cell structure remained. Ultimately, poles were assimilated into spheres as well, although this process occurred more slowly.

We used the fluorescent D-amino acid analogue HADA [23] to visualize changes within the *V*. *cholerae* cell wall during the process of sphere formation. In *V*. *cholerae*, D-amino acids and analogs like HADA can be incorporated into the cell wall via peptide sidestem modification by penicillin-insensitive L,D transpeptidases in the periplasm [23,24]. Thus, at least in *V*. *cholerae*, HADA can be employed as a general cell wall label, even in the presence of cell-wall acting antibiotics.

In antibiotic-free cells, HADA staining was initially distributed evenly over the cell; however, the blebs induced by antibiotics lacked a HADA signal. In contrast, HADA staining was evident in the remainder of the cell for at least 20 min after blebbing commenced, consistent with maintenance of a PG-based cell structure (Fig 1F). Thus, inhibition of cell wall synthesis in *V*. *cholerae* does not result in a sudden and uniform disintegration of the cell wall; instead, it seems likely that locally confined cuts in PG allow for formation of blebs (which are presumed not to contain cell wall material), followed by gradual degradation of the remaining cell wall material. The ability of PG-deficient cells to survive suggests that the inner and outer bacterial membranes may collectively be able to withstand cellular turgor pressure in the absence of support from PG.

To further characterize sphere anatomy, we used fluorescently labeled proteins with previously demonstrated, distinct subcellular localization patterns [25–27]. In spheres, the periplasm was condensed into one compartment (S2A Fig), consistent with loss of PG’s exoskeletal function. The cytoplasm/inner membrane filled most of each sphere but appeared to often be pushed aside by the condensed periplasmic compartment, while the outer membrane was mostly circular. However, the integrity of the outer membrane in spheres appears to be reduced; spheres were highly susceptible to the membrane-acting agents triton X-100 and polymyxin B (S2B Fig), to which El Tor strains of *V*. *cholerae* are normally resistant.

### Neither amidases nor the majority of lytic transglycosylases are necessary for sphere formation

**Fig 2:**
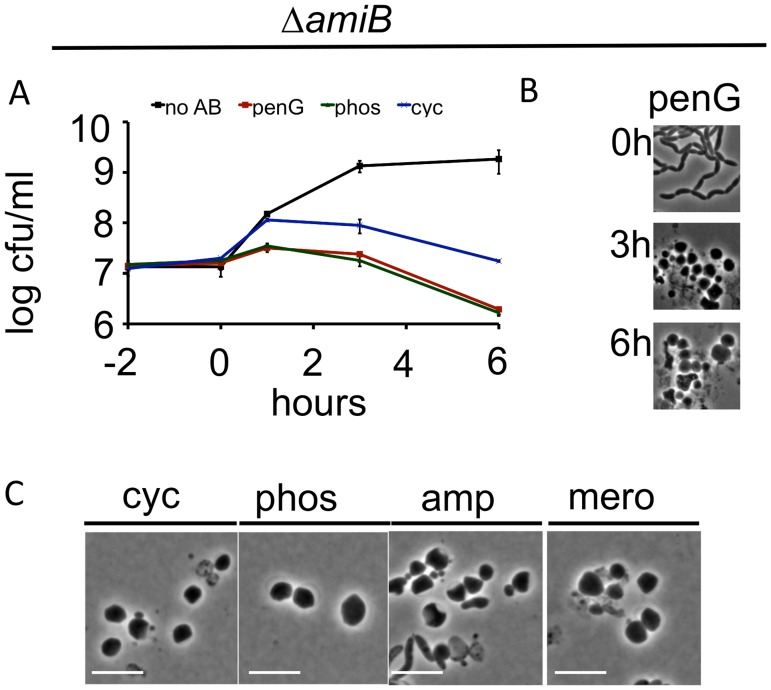
*V*. *cholerae amiB* is not required for sphere formation in response to inhibition of cell wall synthesis. (A-C) A *V*. *cholerae ΔamiB* mutant was treated as described in Fig 1 A-C. Scale bar = 5 μm. No AB = no antibiotic added.

In *E*. *coli*, lysis following exposure to cell-wall acting antibiotics appears to be partially dependent on the redundant PG amidases AmiA, AmiB, and AmiC, whose cleavage of septal PG enables daughter-cell separation [12]. The *V*. *cholerae* genome encodes a single PG amidase (AmiB), and an *amiB* deletion mutant forms long chains of unseparated daughter cells comparable to those of amidase-deficient *E*. *coli* [28]. Unexpectedly, *V*. *cholerae* amidase-deficient cells were more, rather than less, susceptible than wild type cells to killing by penicillin, phosphomycin and D-cycloserine (Fig 2A). However, this susceptibility was not associated with the bacterial lysis seen in drug-treated *E*. *coli*. We observed no significant decrease in culture density (OD_600_) of the *V*. *cholerae amiB* mutant in response to cell-wall acting antibiotics (S3 Fig), and bacterial lysis was rarely observed using light microscopy. Instead, antibiotic treatment ultimately resulted in formation of spherical cells that were similar to those formed by wild type bacteria (Fig 2B and 2C). We speculate that the ultimate decline in viability of the Δ*amiB* mutant under these conditions may reflect its previously noted compromised cell envelope [28]. It is important to note that interpretation of cfu and OD_600_ data is complicated by the fact that the mutant’s multi-cell chains disintegrate into single spheres upon beta lactam treatment (Fig 2B and 2C; discussed below). This is likely the cause of the observed increase in cfu directly after addition of antibiotic in Fig 2A, which may somewhat obscure a loss of viability. The mutant’s increased lag phase compared to the wild type further complicated direct comparisons based on culture density and cfu. Therefore, to directly assess AmiB’s role in the sphere formation process, we turned to single cell analysis.

Time lapse analysis of PenG-induced sphere formation in the Δ*amiB* mutant showed that blebs formed more slowly than in wt cells (compare S4A Fig to Fig 1D), however, we cannot exclude that this is merely due to the decreased growth rate of the Δ*amiB* strain. Moreover, blebbing of Δ*amiB* cells often appeared to originate from outside the midcell (S4A Fig). While the exact location of blebs relative to the septum is difficult to define in this chain-forming mutant, we also noticed blebbing from almost exclusively extraseptal locations when we treated a PBP1A-deficient mutant with the antibiotic cefsulodin, which in *V*. *cholerae* inhibits only PBP1B [26] (S4B Fig and S4C Fig). Since AmiB is presumably active at the septum only [28–30], the occurrence of blebbing outside of the septum in these cells provides additional evidence that enzymes other than AmiB can initiate sphere formation in *V*. *cholerae*. In aggregate, our data suggest that AmiB may play an initiating and facilitating role in sphere formation after inhibition of cell wall synthesis in *V*. *cholerae*, but that other enzymes can partially compensate for its absence.

**Fig 3:**
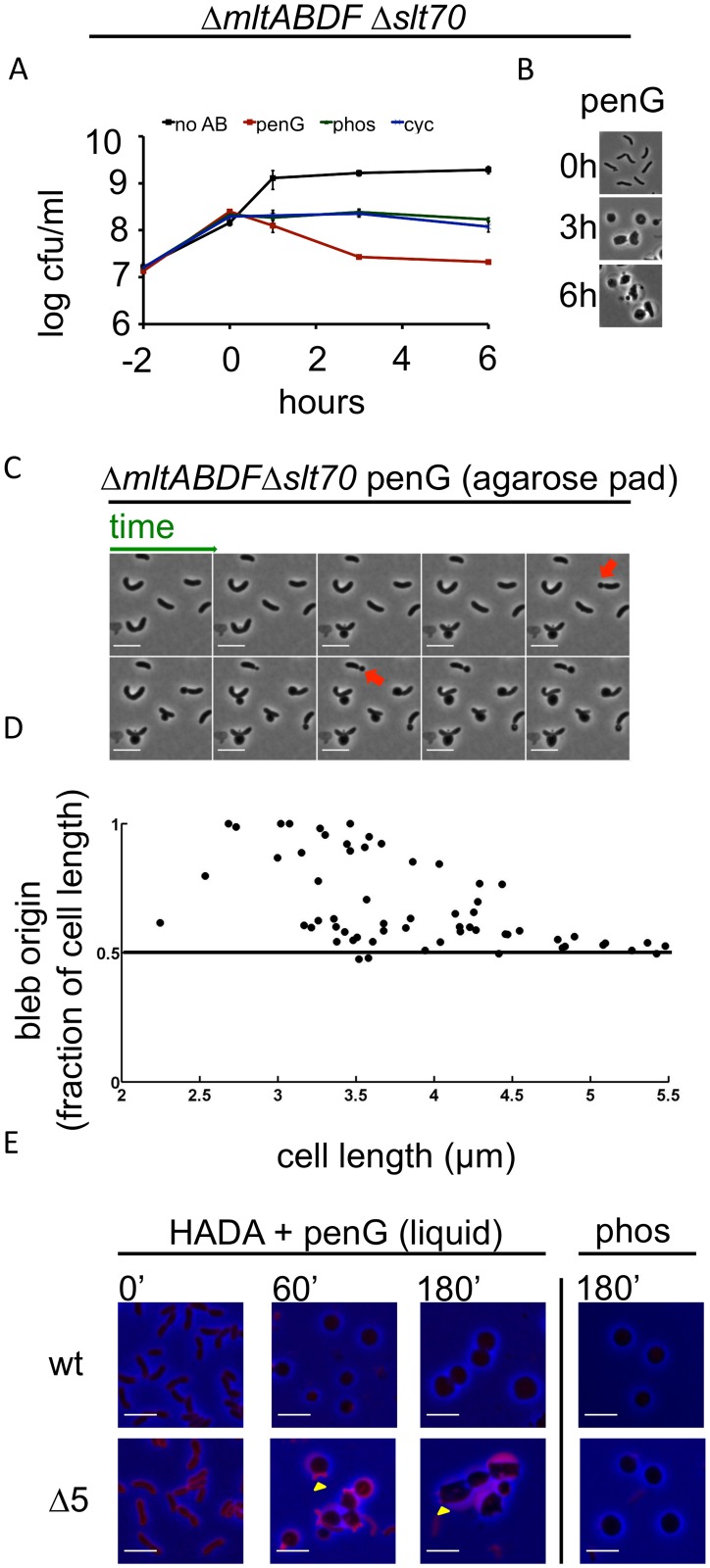
Deletion of multiple lytic transglycosylases alters the kinetics of *V*. *cholerae* sphere formation after inhibition of cell wall synthesis. (A and B) The *Δ5*LTG mutant (Δ*mltABDFΔslt70)* was treated as described in Fig 1A and Fig 1B. No AB = no antibiotic added. (C) Time lapse images of Δ5LTG cells plated on agarose pads containing 100 μg/ml pen G. Frames are 5 min apart, scale bar = 5 μm; red arrowheads point to polar blebs. (D) Quantification of bleb location for time lapses described in C. For details, see Methods. (E) HADA incorporation during exposure to cell wall synthesis inhibitors in broth culture. Cells were grown to exponential phase in the presence of HADA (50 μM), then exposed to 100 μg/ml pen G or phos with HADA remaining in the growth medium. At the indicated time points, samples were washed twice prior to imaging. Images were minimally processed (background subtraction) and are comparable to each other, but not to the one depicted in Fig 1F. Scale bar = 5 μm. Yellow arrowheads point to polar appendages containing PG.

We also assessed the role of lytic transglycosylases (LTGs) in *V*. *cholerae’s* response to antibiotics that inhibit PG synthesis. The *V*. *cholera*e genome encodes 6 predicted LTGs (*mltA*, *mltB*, *mltC*, *mltD*, *mltF*, and *slt70)*. We found that a strain lacking 5 of these (Δ*mltABDF*Δ*slt70*; Δ5LTG) was viable; however, we were unable to obtain a mutant lacking all six. Exposure of Δ5LTG to penicillin resulted in a ~1 log reduction in viability that was not accompanied by lysis, whereas phosphomycin or D-cycloserine did not reduce this strain’s viability (Fig 3A and Fig 3B). Similarly, deletion of certain lytic transglycosylases sensitizes other bacteria to beta lactam antibiotics [13,14], and this has recently been proposed to reflect the LTGs’ role in a quality control mechanism during cell wall synthesis [31].

Penicillin treatment of Δ5LTG or a single disruption of *mltC*, the sixth LTG, yielded spherical cells similar in appearance to those observed after treatment of the wild type strain (Fig 3B and S5 Fig), but the dynamics of sphere formation in Δ5LTG cells were altered. Unlike penicillin-treated wt cells, on agarose pads, a significant proportion (~ 40%) of penicillin-treated Δ5LTG cells started blebbing from sites close to or at the cell poles (Fig 3C and 3D), suggesting that in the absence of LTGs, there is less cleavage of septal PG, consistent with the proposed auxiliary role for these enzymes in cell separation in other bacteria [32,33]. Those cells that initiated blebbing from midcell retained long polar appendages for the entire duration of the experiment (Fig 3C), indicating that the comprehensive disruption of polar and lateral PG observed after beta lactam exposure of wild type cells depends upon one or more LTGs. This was also observed in liquid medium, albeit to a lesser degree, where HADA staining revealed the presence of polar appendages 1–3 hours after exposure to penicillin in Δ5LTG but not in the wt strain (Fig 3E). Additionally, HADA stained Δ5LTG cells much more intensely than wt cells before exposure to penicillin (Fig 3E and S6 Fig) and the mutant cells also retained more fluorescent material in the periplasm during exposure to the antibiotic. Thus, the Δ5LTG mutant appears to have reduced PG turnover, a deficiency that is accentuated by exposure to beta lactam antibiotics. Since beta lactams inhibit the transpeptidase activity of PBPs, but presumably leave their transglycosylase activity intact, it is possible that the periplasmic accumulation of HADA stain in the Δ5LTG mutant reflects the build-up of minimally cross linked (and HADA-labeled) PG strands that would ordinarily be degraded by lytic transglycosylases. Consistent with this possibility, we found that neither the Δ5LTG mutant nor wt cells treated with phosphomycin (an antibiotic that affects precursor synthesis and thus should not allow any PG synthesis) accumulated HADA-labeled material (Fig 3E). In summary, lytic transglycosylases, at least MltABDF and Slt70 in combination, or MltC alone, are not required for sphere formation but appear to be involved in downstream processes of cell wall degradation.

### An endopeptidase enables sphere formation

**Fig 4:**
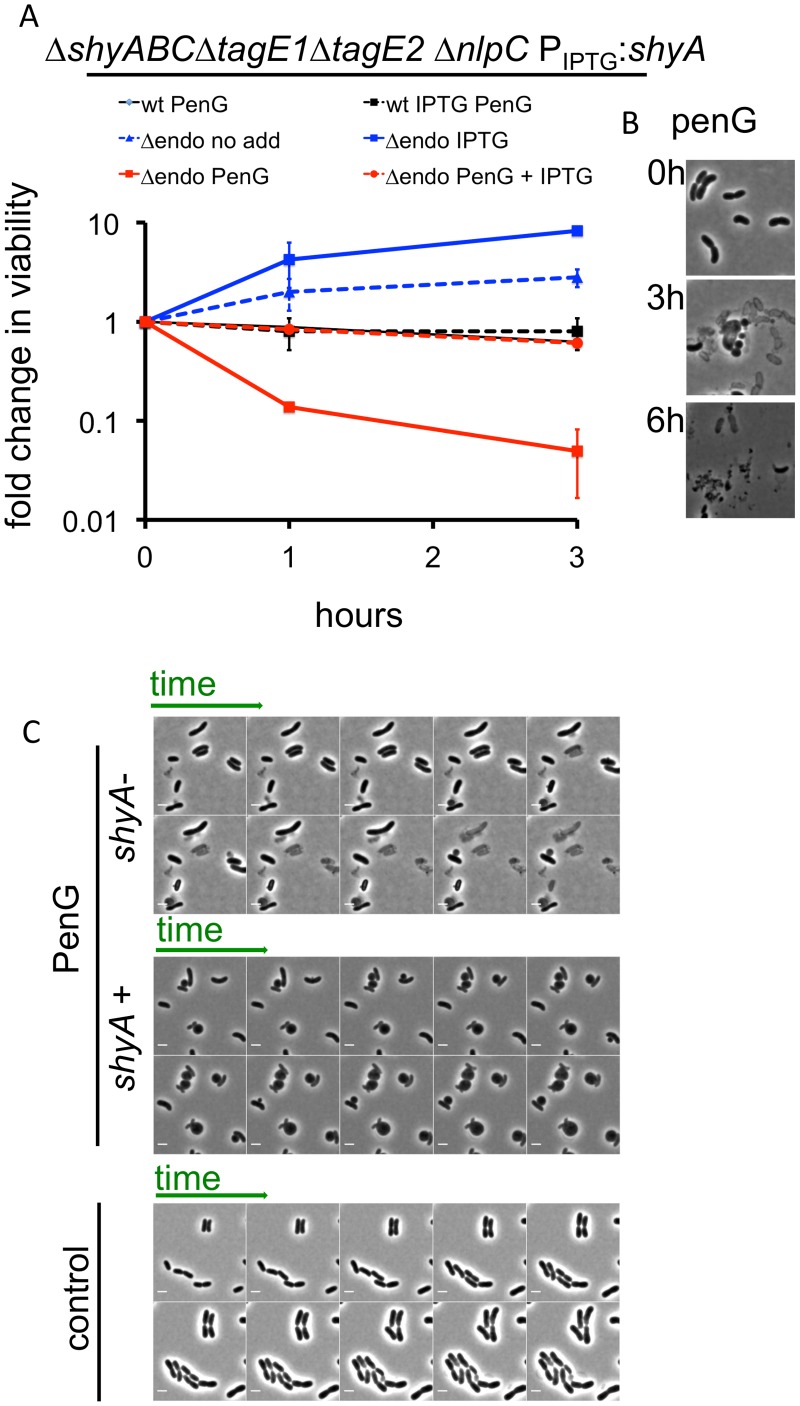
*V*. *cholerae* endopeptidase are required to prevent lysis in response to inhibition of cell wall synthesis. (A) Wild type *V*. *cholerae* and a Δendo (Δ*shyABC* Δ*nlpC* Δ*tagE1* Δ*tagE2* P_tac_:*shyA*) mutant were grown in the presence or absence of 200 μM IPTG for 1.5 h (= T0) and then exposed to pen G. Cfu/ml at the indicated time points were normalized to cfu/ml at T0 and shown as fold change in viability. Data are mean of two biological replicates; error bars represent standard deviation. (B) Representative images of *V*. *cholerae* Δendo cells at different time points after exposure to penicillin G from the experiment shown in (A). (C) Time lapse images of Δendo cells initially grown for 1.5 h in either the presence or absence of IPTG (25 μM) and subsequently imaged on agarose pads containing 100 μg/ml pen G. The pad for ShyA+ cells also contained IPTG. The control panel depicts cells applied to an agarose pad containing neither antibiotic nor IPTG. Frames in the lower (control) panel are 10 min apart; in the other panels, frames are 5 min apart. Scale bar = 2 μm.

Since amidases and the 5 LTGs were not critical for sphere formation, we turned our focus towards endopeptidases, two of which (ShyA and ShyC) are synthetically lethal and essential for cell elongation in *V*. *cholerae* [25]. The *V*. *cholerae* genome encodes five periplasmic M23 endopeptidases and one P60 family endopeptidase (NlpC). We constructed a mutant that lacks all predicted non-PBP endopeptidases and expresses inducible *shyA* from a neutral chromosomal locus (Δ*shyA* Δ*shyB* Δ*shyC* Δ*nlpC* Δ*tagE1* Δ*tagE2* P_tac_:*shyA*; Δendo). In this background, depletion of *shyA* slowed growth, similar to our previous observations with a Δ*shyA* Δ*shyC* P_tac_:*shyA* strain, which is defective in cell elongation but proficient in cell division [25](Fig 4A). Importantly, untreated Δendo cells did not lyse even after extended ShyA depletion (Fig 4C and “control”). Unexpectedly, however, exposure of ShyA-depleted Δendo cells to penicillin G resulted in rapid loss of viability and concomitant lysis of the majority of the population (Fig 4A and 4B). Lysis could be prevented and sphere formation restored by expression of *shyA* (Fig 4C). A similar pattern was observed with D-cycloserine and phosphomycin (S7 Fig). Notably, analysis of the dynamics of cell lysis using time lapse microscopy revealed that cell disintegration did not proceed through a spherical intermediate (Fig 4C). These results suggest that, paradoxically, the presence of a D,D endopeptidase (ShyA), a putative ‘autolysin’, prevents lysis and enables formation of viable spheres after exposure of *V*. *cholerae* to a beta lactam antibiotic.

The lysis phenotype was also observed in a ShyA-depleted Δ*shyA* Δ*shyB* Δ*shyC* P_tac_:*shyA* (S8 Fig) strain, and expression of ShyC but not ShyB could at least partially prevent lysis of ShyA-depleted Δendo (S8 Fig), demonstrating that either one of the paralogues ShyA and ShyC must be present for beta lactam tolerance and formation of viable spheres, while the other M23 endopeptidases and NlpC are dispensable. We also observed penicillin G-induced lysis of Δendo Δ*amiB* cells when ShyA was depleted (S8 Fig), as well as in Δendo cells with additional mutations in LTG genes (Δendo Δ*mltB*Δ*mltD* and Δendo Δ*mltB* Δ*slt70*, which were the only strains with multiple LTG disruptions that we were able to make in the Δendo background). These results indicate that neither amidase activity nor MltB, MltD or Slt70 activity are necessary to cause lysis of Δendo cells. Lastly, ShyA-depleted Δendo cells were not more susceptible to membrane-acting agents than ShyA-replete cells (S9 Fig), suggesting that the observed lysis phenotype is not simply the consequence of a general weakness of the cell envelope.

**Fig 5:**
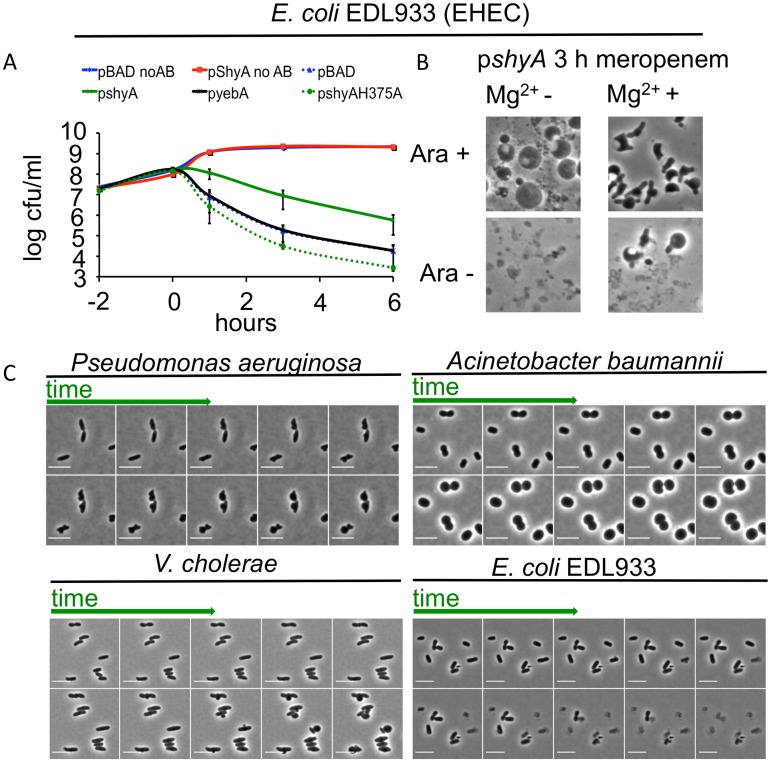
Inhibition of cell wall synthesis in other bacteria does not always lead to cell lysis. (A) Influence of *shyA* or *yebA* overexpression on viability of *E*. *coli* EDL933 treated with meropenem. EDL933 carrying either pBAD33 (plasmid vector) or its derivatives encoding *yebA* or *shyA* was grown to exponential phase in the presence of arabinose, then meropenem (1 μg/ml) was added at (T0) and viable cell counts determined at the indicated times. Data are average of three independent experiments; error bars represent standard deviation. (B) Representative images from the 3 h time point of an experiment similar to the one depicted in (A), with or without the addition of 10 mM Mg^2+^ in the growth medium. (C) Time lapse images of *P*. *aeruginosa*, *A*. *baumannii*, *V*. *cholerae* and EDL933 cells exposed to meropenem. Cells were grown in LB until OD_600_ ~ 0.5 and then applied to an agarose pad (10% LB/PBS) containing meropenem (4 μg/ml) as well as MgSO_4_ (10 mM)/CaCl_2_ (1 mM) for *P*. *aeruginosa* and *A*. *baumannii* only. Frames are 9 min apart, scale bar = 5 μm. No AB = no antibiotic added.

Since ShyA could prevent beta lactam-mediated lysis in *V*. *cholerae*, we tested whether it could protect a heterologous organism from lysis after inhibition of cell wall synthesis. We overproduced ShyA in the EHEC isolate EDL933 and measured its survival after exposure to meropenem. Overexpression of *shyA* alone did not influence EHEC growth. However, expression of this endopeptidase increased EHEC’s capacity to survive meropenem exposure by ~10-fold compared to an empty vector control, overexpression of *yebA*, *E*. *coli’s* ShyA homologue (Fig 5A) or overexpression of ShyA carrying an active site mutation (H375A). Meropenem-treated EDL933 expressing ShyA formed spheres, while the control cells carrying the empty vector rapidly lysed (Fig 5B). These data suggest that ShyA activity is linked to survival in the presence of beta lactam antibiotics, and that ShyA-mediated cleavage of the cell wall may differ from YebA-mediated cleavage events, although it is theoretically possible that these results only reflect differences in the expression levels for the two proteins.

When EDL933 was grown in the presence of Mg^2+^, which is thought to have a stabilizing effect on the outer membrane [11], cells carrying an empty (control) plasmid formed spheres at a low but detectable frequency after meropenem exposure. However, in this medium, visible lysis was still markedly reduced and sphere formation concomitantly enhanced after overexpression of plasmid-encoded *shyA* (Fig 5B). ShyA’s protective effect can thus apparently be enhanced by additional stabilization of the outer membrane, suggesting that multiple processes can contribute to beta lactam tolerance.

Finally, a recent report showed that *Pseudomonas aeruginosa* could assume a spherical morphology in response to carbapenem antibiotics, especially in media fortified with Mg^2+^ and Ca^2+^ [34], suggesting that the absence of cell lysis after inhibition of cell wall synthesis may not be limited to *V*. *cholerae*.

*P*. *aeruginosa* turned into spheroid forms on agarose pads containing meropenem via steps that resemble *V*. *cholerae’s* transformation into spheres after exposure to this antibiotic (Fig 5C), suggesting that the mechanisms underlying sphere formation may be similar in these bacteria. Notably, we observed that under the same experimental conditions, the multidrug resistant *Acinetobacter baumannii* clinical isolate Lac-4 also failed to lyse in response to meropenem, albeit in a process unlike that observed in *V*. *cholerae* and *P*. *aeruginosa* (Fig 5C). Thus, population-wide lysis in response to inhibition of cell wall synthesis may not be the norm for many bacteria.

### Conclusions

In summary, we found that inhibition of cell wall synthesis does not inexorably lead to cell lysis and death, as occurs in the model organism *E*. *coli*. Some pathogenic bacteria survive blockade of PG synthesis, and instead form viable spheres. Surprisingly, in *V*. *cholerae*, sphere formation/survival depends on the activity of PG hydrolases (autolysins), particularly the D,D endopeptidase ShyA. Thus, our findings suggest that although the pathways and enzymes that mediate PG degradation after inhibition of PG synthesis are critical for determining bacterial fate under these conditions, such fates are variable: in different organisms, enzymes that ordinarily degrade PG can either lead to lysis or promote survival when PG synthesis is blocked by antibiotics.

In *V*. *cholerae*, our data suggests that there is an ordered series of steps that lead to sphere formation following interference with cell wall synthesis. In the majority of cells, the first cell wall lesion from which blebbing commences is likely generated by AmiB, perhaps with some assistance from LTGs. The latter enzymes play a more critical role in downstream processes that lead to PG resorption. The procession from blebs to spheres rather than lysis requires the presence of an endopeptidase, either ShyA or ShyC. Our findings suggest that the specificity, rate or location of PG hydrolysis mediated by such D,D endopeptidases is important for preventing cell lysis; however, the exact mechanism by which these proteins prevent lysis and death requires further investigation. It is tempting to speculate that while the end result of cell wall synthesis inhibition may differ between bacteria, the hierarchy of cell wall lytic events might be conserved.

It will be interesting to explore whether PG-degrading enzymes important for cell elongation are required for sphere formation in other organisms, as in *V*. *cholerae*. Our observations suggest that the absence of lysis after treatment with beta lactam antibiotics may be more common than currently appreciated, but the determinants of such survival have not been identified. Importantly, some reports have suggested that spherical bacteria can be isolated from patients treated with beta lactam antibiotics during chronic infections (e.g. respiratory infections caused by *Haemophilus influenzae*, [35]). Thus, similar to persister cells [19], population-wide tolerance and sphere formation may represent another fairly widespread way by which bacteria can evade the lethal consequences of beta lactam exposure. New antibiotics that target processes critical for sphere formation (e.g. inhibitors of ShyA) or for sphere survival might exhibit potent synergy with beta lactams and thus provide a novel approach for improved antimicrobial therapeutics.

## Materials and Methods

### Strain construction

Gene deletions were conducted by standard techniques using suicide plasmid (pCVD442) containing ~600 bp flanking regions of the gene to be deleted [25]. All knockouts are substitutions of the respective open reading frame with the linker sequence 5’-TTATCATTACTCGAGTGCGGCCGCATGAAA-3’.

Overexpression plasmids were constructed by amplifying the gene of interest including its native ribosome binding site and cloning into Sma1-digested pBAD33 using isothermal assembly [36].

### Growth and killing experiments

Cells were grown in LB medium at 37°C. Growth curves were conducted in 200 μL volume in 200 well honeycomb plates using a Biotek growth curve machine.

For time-dependent killing experiments, overnight cultures were diluted 1: 100 into 3 ml LB medium and grown shaking at 37°C until cell density reached ~ 2 x 10^8^ cfu/ml (~OD_600_ 0.3). Antibiotics (Sigma) were added to either 100 μg/ml (penicillin G, ampicillin, phosphomycin, D-cycloserine, cefsulodine) or 10 μg/ml (meropenem). At the indicated time points, cells were serially diluted and spot plated to determine cfu/ml.

For post-penicillin survival assays, cells grown as described above were exposed to penicillin G for three hours; then either nothing, Triton X-100 (1% final concentration) or Polymixin B (40 μg/ml final concentration) were added, followed by 30 min incubation at 37°C and subsequent spot-plating for cfu/ml.

For antibiotic exposure assays in cecal fluid, 200 μL of cecal fluid from infected infant rabbits (which contains a high-density monoculture of *V*. *cholerae*, [37]) was collected ~ 16 h post infection, transferred to eppendorf tubes and incubated standing at 37°C for 3 h after addition of antibiotic.

For depletion experiments, overnight cultures of Δendo were diluted 1: 100 into 3 ml LB medium containing 25 μM IPTG (which is the minimal growth permitting IPTG concentration). These cultures were then grown for 2 h, washed 2 x with fresh medium and then resuspended in LB lacking IPTG and grown for an additional 1.5 h. Cells were then directly applied to agarose pads (see below) containing penicillin G with or without IPTG (100 μM).

### MIC determination

For assessment of minimum inhibitory concentrations (MIC), overnight cultures were diluted 1000fold into fresh LB medium, grown for 1 h and again diluted 1:1000 in fresh LB medium. 50 μL of this inoculum were applied to 96 well plates containing 50 μL of 2fold serial dilutions of the antibiotic to be tested. The MIC was read as the lowest antibiotic concentration at which no turbidity was visible.

### Image acquisition and analysis

Time lapse experiments were conducted on 0.8% agarose pads with 10% LB and PBS. Images were analyzed using ImageJ software, and adjusted by removing background fluorescence (using imageJ’s built-in function, 50 px rolling ball radius) and adjusting brightness/contrast levels where appropriate (i.e. in Fig 1F). Care was taken to use the same adjustment parameters for images that were to be compared directly with each other (i.e. in Fig 3E, wt vs. Δ5).

## Supporting Information

S1 Fig *V*. *cholerae* averts penicillin-induced death and lysis. (A) Concentration-dependent survival. *V*. *cholerae* cultures were treated with increasing concentrations of penicillin G (MIC = 5 μg/ml) for 3h and survival measured by spot-plating. (B)Twenty hours after infant rabbits were infected with *V*. *cholerae* (35), cecal fluid containing ~ 10^8^ cfu/ml *V*. *cholerae* cells was collected, and penicillin (100 μg/ml, 20 x MIC) was added; after 3 h cells were imaged.
(TIFF)

S2 Fig Anatomy and susceptibility pattern of spheres. (A) Microscopy-based detection of markers of subcellular components in meropenem-induced spheres; markers included: outer membrane, LpoA-mCherry; inner membrane, YFP-PBP1A; periplasm, CsiV-mCherry; cytoplasm, cytoplasmic GFP. (B) Marked reduction in viability of penicillin G-induced spheres after exposure to Triton X-100 (1%) or to polymyxin B (40 μg/ml) compared with non-antibiotic treated exponential phase (EP) or stationary phase (SP) cells. Wt cells were exposed to penicillin G (100 μg/ml) for 3 h before treatment with Triton X-100 or polymyxin B.
(TIFF)

S3 Fig Δ*amiB* cells do not lyse in the presence of penicillin G. Penicillin G was added at 0 h. Graph represents averages of two biological replicates. Errors bars represent standard deviation.
(TIFF)

S4 Fig Inhibition of cell wall synthesis in Δ*amiB* and Δ*pbp1a V*. *cholerae* treated with cefsulodin leads to sphere formation through extraseptal blebbing. (A) Time lapse images of Δ*amiB* cells plated on an agarose pad containing 100 μg/ml pen G. Frames are 5 min apart, scale bar = 5 μm. A constitutive, cytoplasmic GFP (false-colored in red) was used to allow detection of single cell boundaries. (B) Time lapse images of *V*. *cholerae* Δ*pbp1a* grown in the presence of cefsulodin, which inhibits Pbp1b (23). Frames are 5 min apart. (C) Analysis of locations of sites of bleb initiation as described in legend to Fig 1E. Scale bar = 5 μm.
(TIFF)

S5 Fig An *mltC* insertion mutant forms spheres in response to penicillin G exposure. The *mltC*::Tn strain was grown to exponential phase and exposed to penicillin G for 3 h.
(TIFF)

S6 Fig HADA stains the cell wall of Δ5LTG more intensely than wild type. Exponential phase cells were exposed to 50 μM HADA for 30 min, washed twice and imaged. Images were analyzed using MicrobeTracker and Matlab. Shown are histograms of total fluorescence normalized to cell size. Vertical lines (red for wt, blue for Δ5LTG) represent mean fluorescence intensities
(TIFF)

S7 Fig Diverse inhibitors of cell wall synthesis induce lysis in ShyA-depleted Δendo. Δendo and wt cultures were grown for 1.5 h in either the presence or absence of 200 μM IPTG (to induce ShyA expression) prior to exposure to (A) D-cycloserine (100 μg/ml) or (B) fosfomycin (100 μg/ml) (= T0). OD_600_ kinetics were assessed in a microplate OD reader. Graph represents averages of one experiment done in technical quadruplicates and representative of two independent experiments with similar results.
(TIFF)

S8 Fig Lysis of Δendo derivatives in penicillin G. All strains were treated and imaged as described for Fig 4C except for Δendo derivatives carrying pBAD*shyC* or pBAD*shyB*, which were grown in the presence of arabinose to induce expression of *shyC* or *shyB* respectively.
(TIFF)

S9 Fig A multiple endopeptidase knockout is not susceptible to membrane damage *per se*. ShyA was depleted from Δendo as described in the legend for Fig 4, followed by 30 min exposure to 1% bile, 100 μg/ml penicillin G (PenG) or 0.1% SDS. Log percent survival is cfu/ml after 30 min normalized to initial cell count. Values shown are averages of two independent experiments; error bars represent standard deviation.
(TIFF)
