# Supplementary figures and images for: Endopeptidase-Mediated Beta Lactam Tolerance
Source: PLoS Pathog. 2015 Apr 17;11(4):e1004850. doi: 10.1371/journal.ppat.1004850 (PMC4401780; doi:10.1371/journal.ppat.1004850)

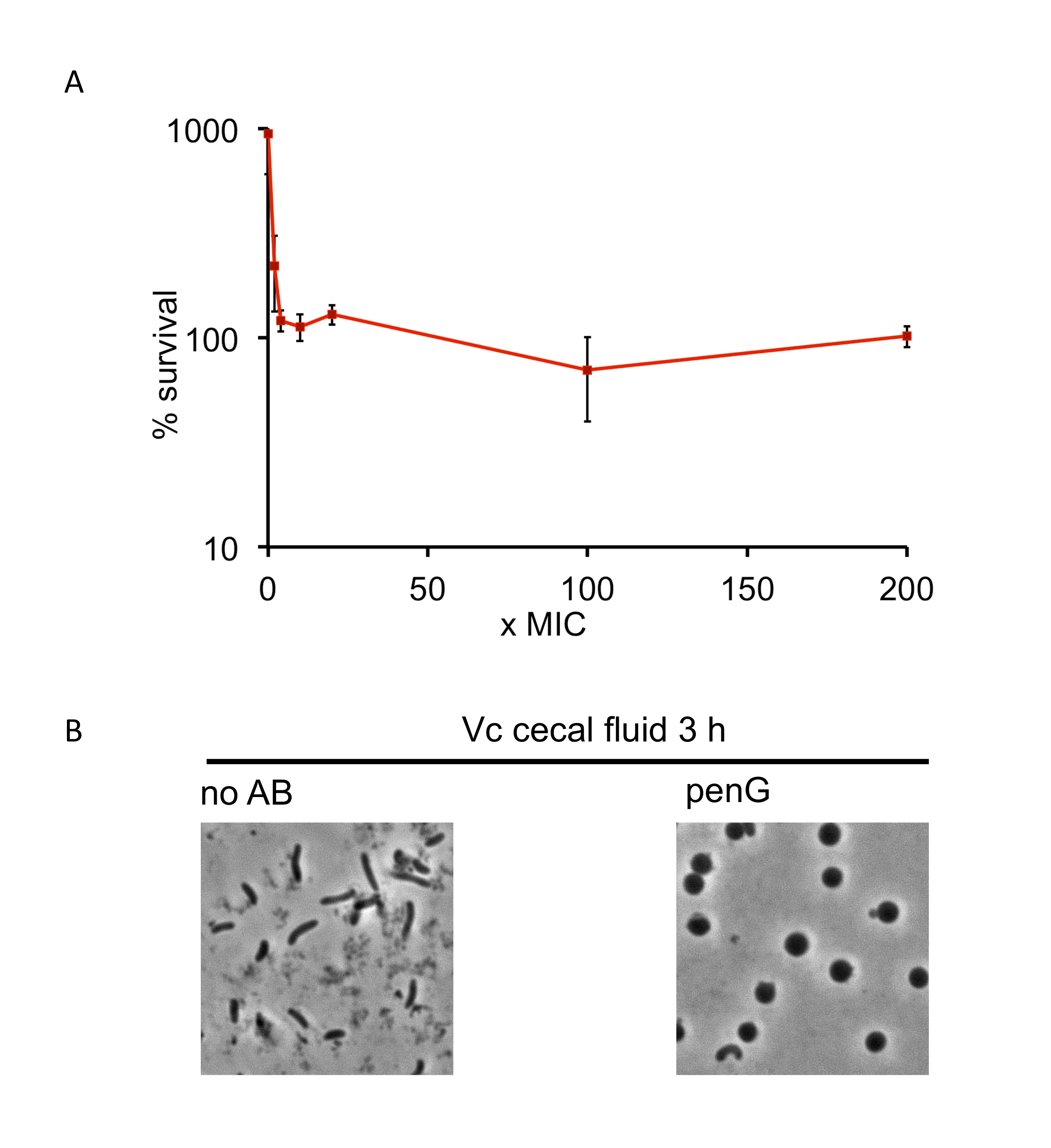

Supplement: S1 Fig — (A) Concentration-dependent survival. V. cholerae cultures were treated with increasing concentrations of penicillin G (MIC = 5 μg/ml) for 3h and survival measured by spot-plating. (B)Twenty hours after infant rabbits were infected with V. cholerae (35), cecal fluid containing ~ 108 cfu/ml V. cholerae cells was collected, and penicillin (100 μg/ml, 20 x MIC) was added; after 3 h cells were imaged. (TIFF) [file ppat.1004850.s001.tiff]

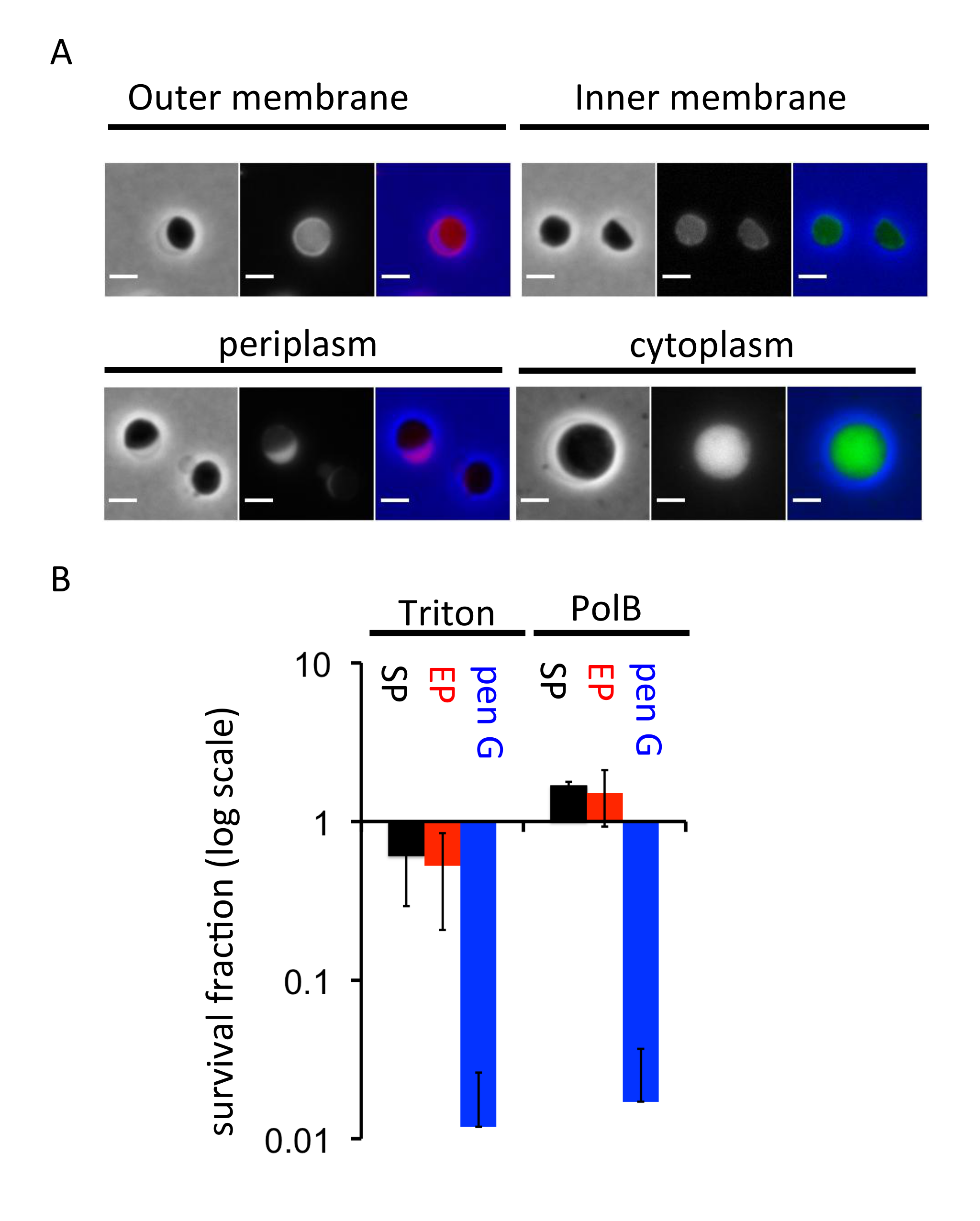

Supplement: S2 Fig — (A) Microscopy-based detection of markers of subcellular components in meropenem-induced spheres; markers included: outer membrane, LpoA-mCherry; inner membrane, YFP-PBP1A; periplasm, CsiV-mCherry; cytoplasm, cytoplasmic GFP. (B) Marked reduction in viability of penicillin G-induced spheres after exposure to Triton X-100 (1%) or to polymyxin B (40 μg/ml) compared with non-antibiotic treated exponential phase (EP) or stationary phase (SP) cells. Wt cells were exposed to penicillin G (100 μg/ml) for 3 h before treatment with Triton X-100 or polymyxin B. (TIFF) [file ppat.1004850.s002.tiff]

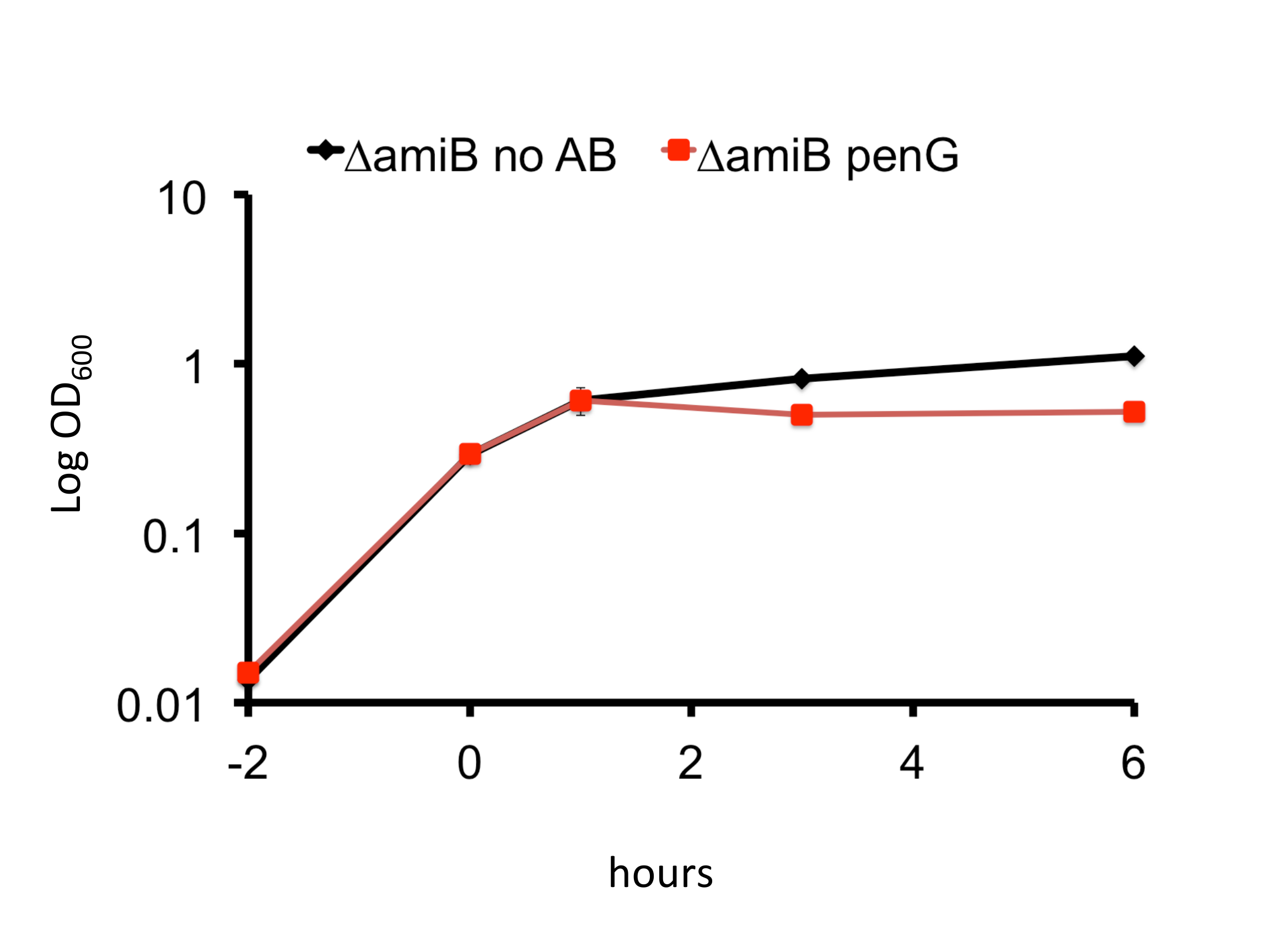

Supplement: S3 Fig — Penicillin G was added at 0 h. Graph represents averages of two biological replicates. Errors bars represent standard deviation. (TIFF) [file ppat.1004850.s003.tiff]

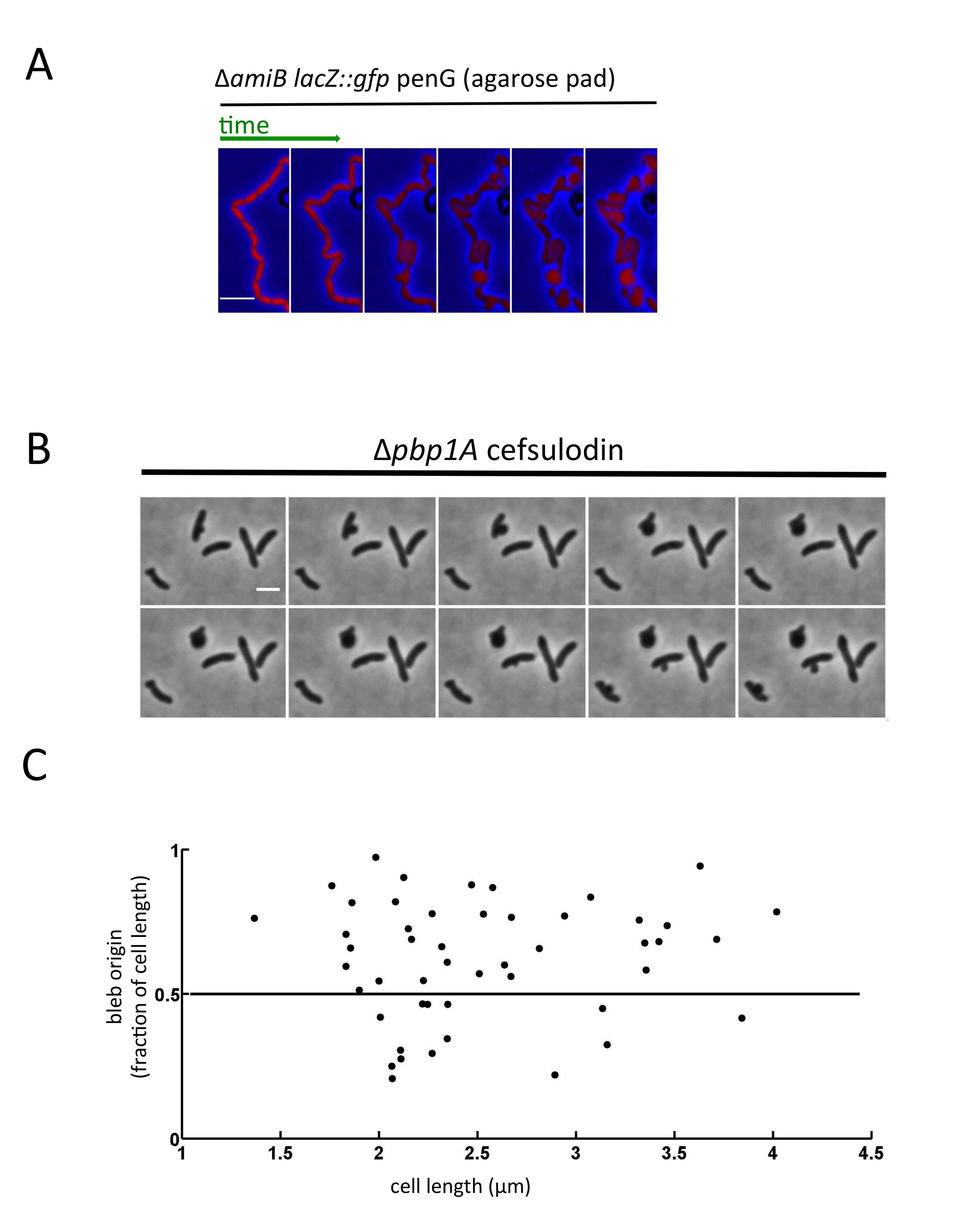

Supplement: S4 Fig — (A) Time lapse images of ΔamiB cells plated on an agarose pad containing 100 μg/ml pen G. Frames are 5 min apart, scale bar = 5 μm. A constitutive, cytoplasmic GFP (false-colored in red) was used to allow detection of single cell boundaries. (B) Time lapse images of V. cholerae Δpbp1a grown in the presence of cefsulodin, which inhibits Pbp1b (23). Frames are 5 min apart. (C) Analysis of locations of sites of bleb initiation as described in legend to Fig 1E. Scale bar = 5 μm. (TIFF) [file ppat.1004850.s004.tiff]

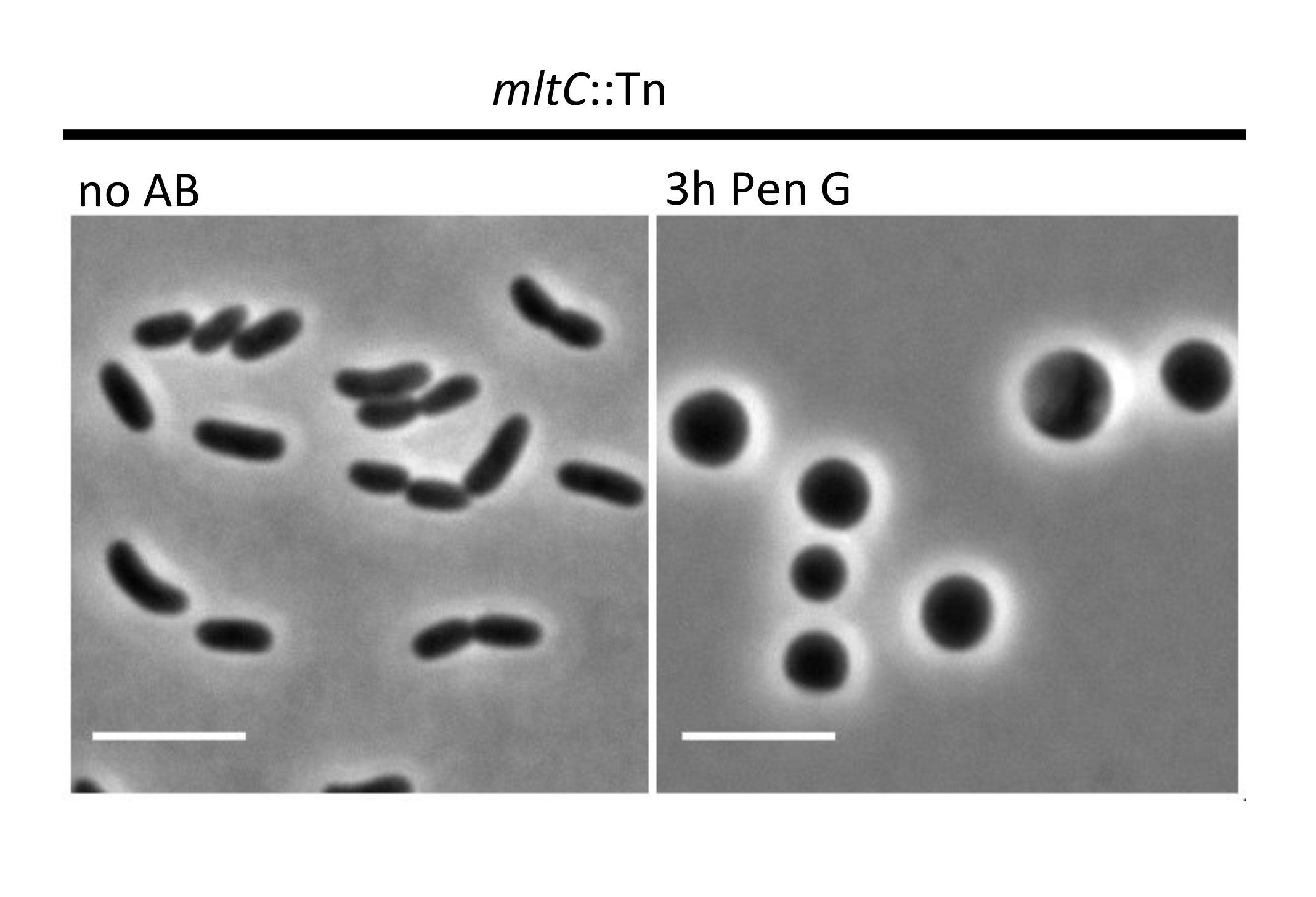

Supplement: S5 Fig — The mltC::Tn strain was grown to exponential phase and exposed to penicillin G for 3 h. (TIFF) [file ppat.1004850.s005.tiff]

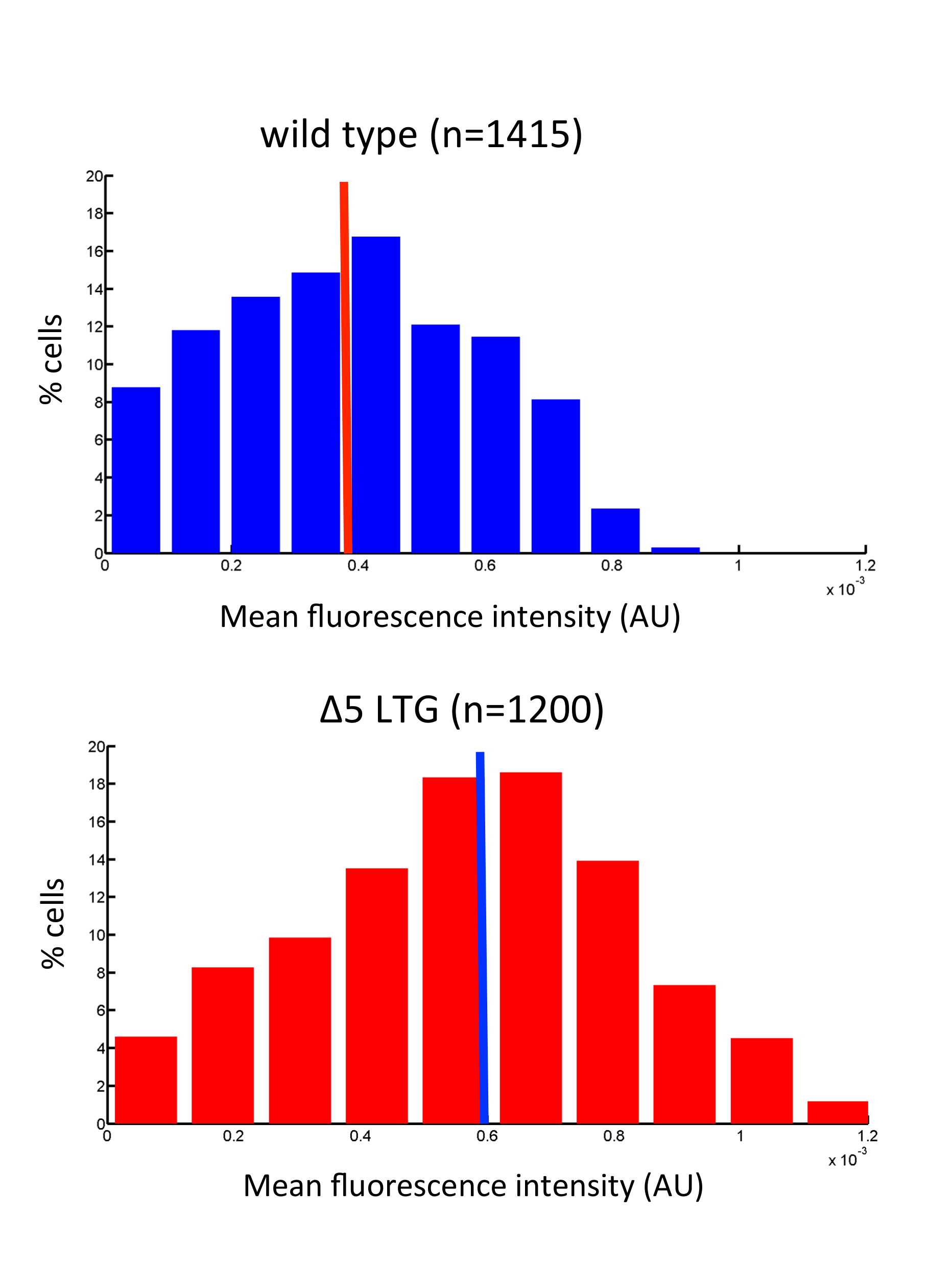

Supplement: S6 Fig — Exponential phase cells were exposed to 50 μM HADA for 30 min, washed twice and imaged. Images were analyzed using MicrobeTracker and Matlab. Shown are histograms of total fluorescence normalized to cell size. Vertical lines (red for wt, blue for Δ5LTG) represent mean fluorescence intensities (TIFF) [file ppat.1004850.s006.tiff]

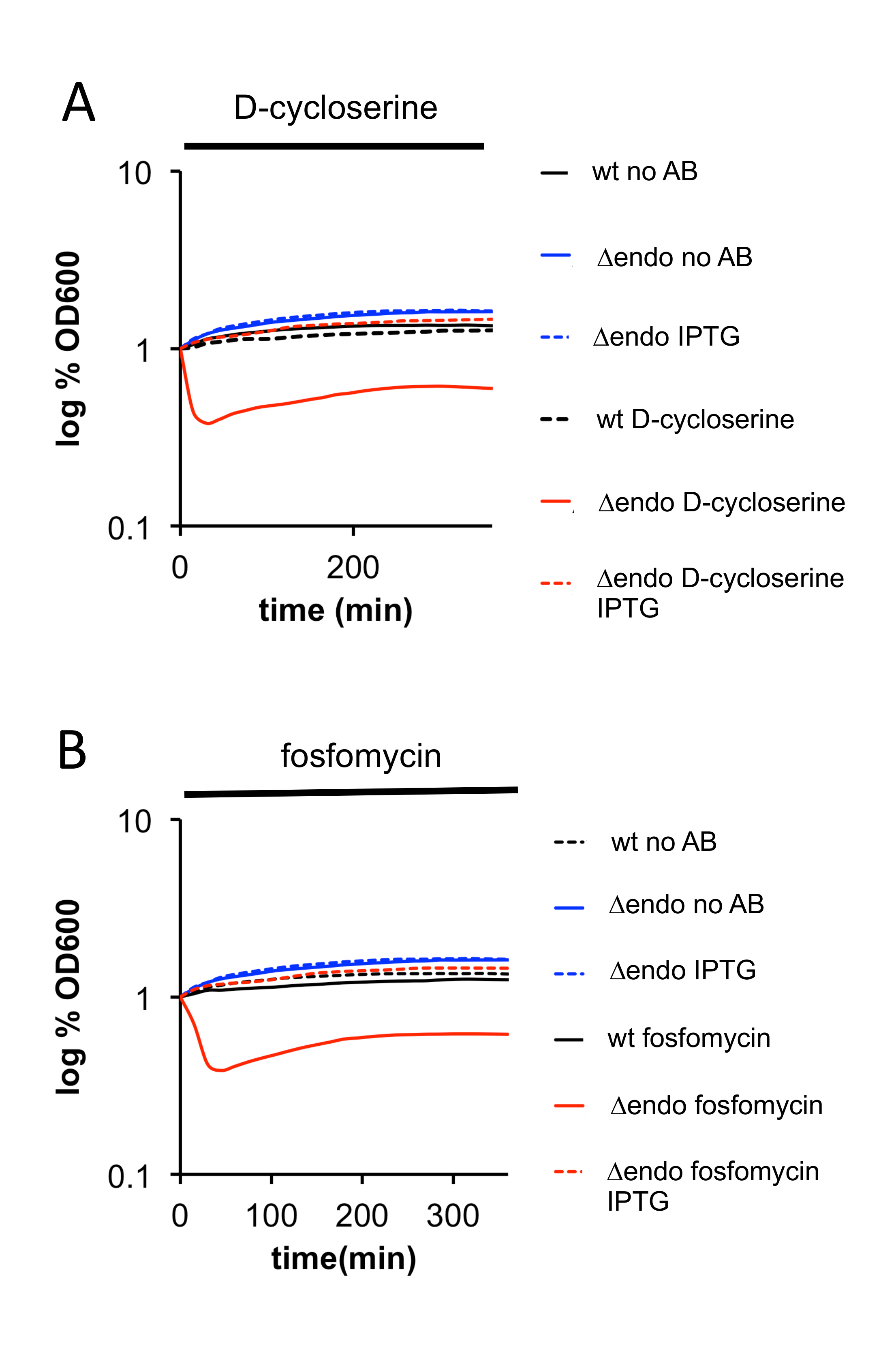

Supplement: S7 Fig — Δendo and wt cultures were grown for 1.5 h in either the presence or absence of 200 μM IPTG (to induce ShyA expression) prior to exposure to (A) D-cycloserine (100 μg/ml) or (B) fosfomycin (100 μg/ml) (= T0). OD600 kinetics were assessed in a microplate OD reader. Graph represents averages of one experiment done in technical quadruplicates and representative of two independent experiments with similar results. (TIFF) [file ppat.1004850.s007.tiff]

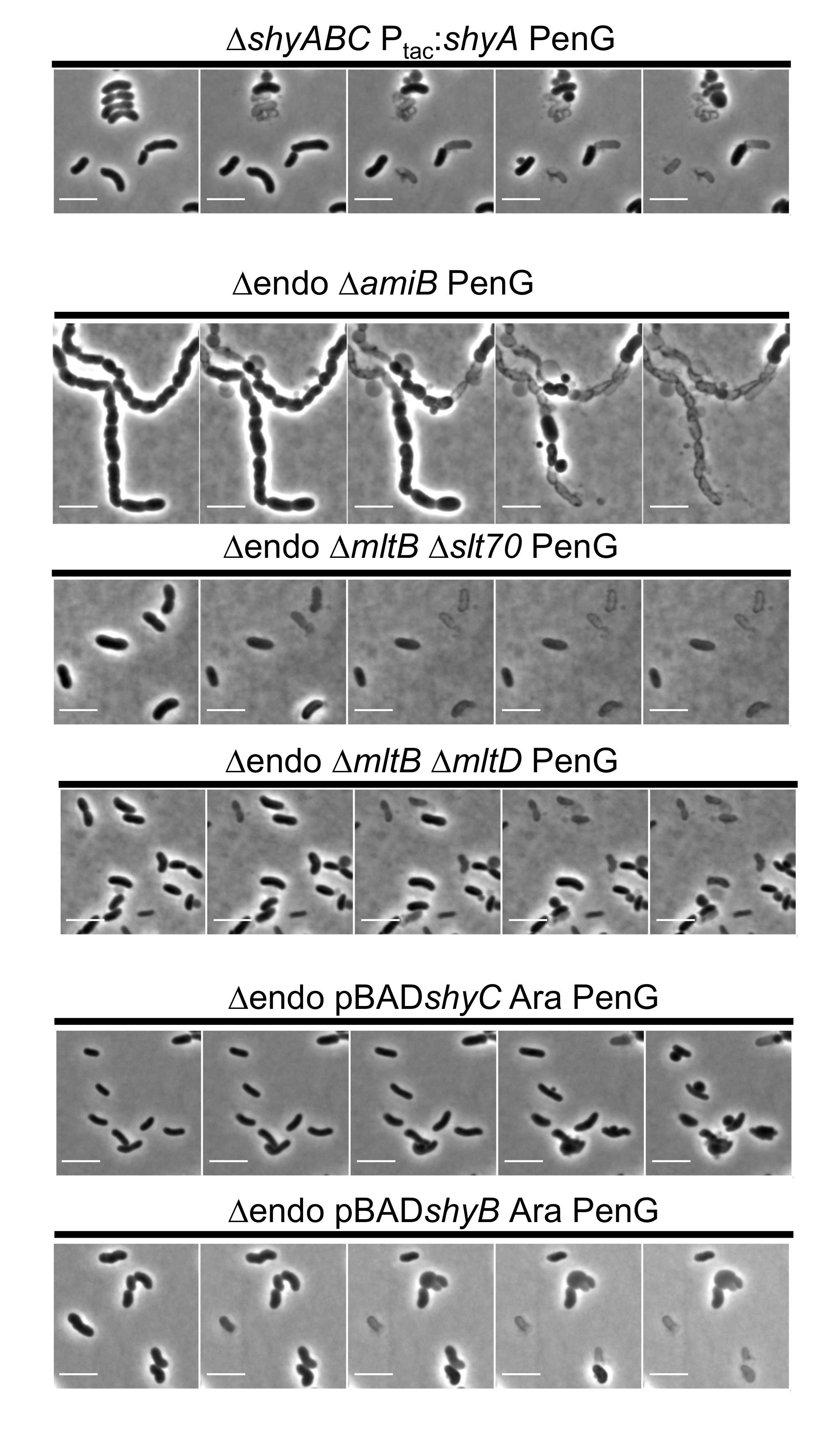

Supplement: S8 Fig — All strains were treated and imaged as described for Fig 4C except for Δendo derivatives carrying pBADshyC or pBADshyB, which were grown in the presence of arabinose to induce expression of shyC or shyB respectively. (TIFF) [file ppat.1004850.s008.tiff]

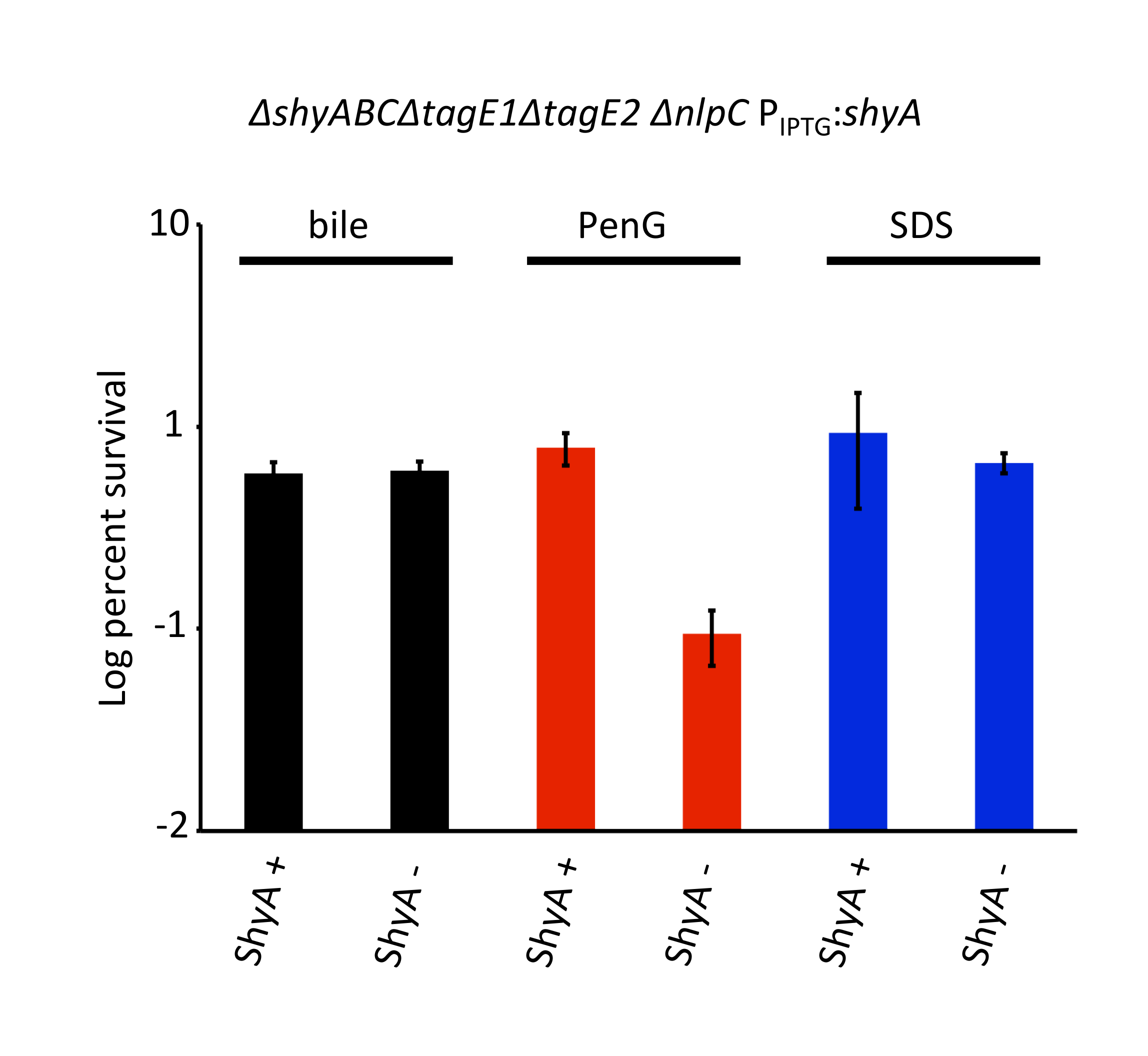

Supplement: S9 Fig — ShyA was depleted from Δendo as described in the legend for Fig 4, followed by 30 min exposure to 1% bile, 100 μg/ml penicillin G (PenG) or 0.1% SDS. Log percent survival is cfu/ml after 30 min normalized to initial cell count. Values shown are averages of two independent experiments; error bars represent standard deviation. (TIFF) [file ppat.1004850.s009.tiff]
